# From genes to function: regulation, maturation, and evolution of cytochrome *c* nitrite reductase in nitrate reduction to ammonium

**DOI:** 10.1128/aem.00292-25

**Published:** 2025-06-09

**Authors:** Krystina Hird, Julius O. Campeciño, Eric L. Hegg

**Affiliations:** 1 Department of Biochemistry & Molecular Biology, Michigan State University 123744 , East Lansing, Michigan, USA; Georgia Institute of Technology, Atlanta, Georgia, USA

**Keywords:** NrfA, NrfH, cytochrome *c *nitrite reductase, dissimilatory nitrate reduction to ammonium, NrfBCD, gene regulation, quinol oxidase

## Abstract

The dissimilatory nitrate reduction to ammonium pathway converts nitrate to ammonium, a vital reaction in the global nitrogen cycle. The second step of the pathway is performed by cytochrome *c* nitrite reductase (NrfA), a soluble, periplasmic cytochrome responsible for the reduction of nitrite to ammonium. The pentaheme NrfA catalyzes this six-electron and eight-proton reduction of nitrite at a single active site with the help of its quinol oxidase partners. In this review, we discuss our current understanding of (i) the structure, homology, and evolution of both NrfA and its redox partners, (ii) the regulation of the *nrf* operon, and (iii) the maturation of NrfA proteins via unique cytochrome maturation pathways.

## INTRODUCTION

**Fig 1:**
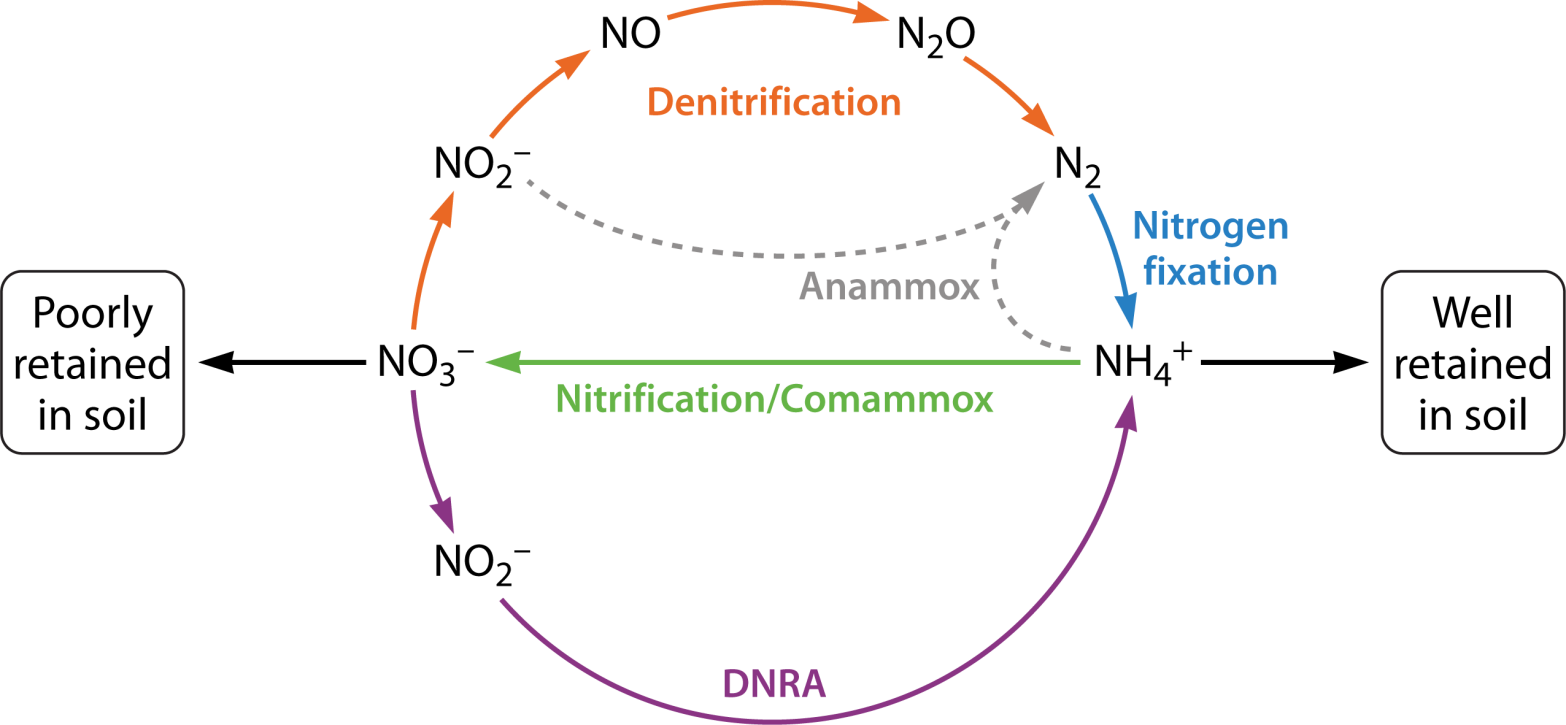
Schematic representation of the nitrogen fixation, nitrification/comammox, anammox, denitrification, and dissimilatory nitrate reduction to ammonium (DNRA) pathways. Due to space limitations, not all intermediates are shown. In particular, hydroxylamine (NH_2_OH), which is formed during both aerobic and anaerobic ammonia oxidation, and hydrazine, whose production has been exclusively found in anammox bacteria, are not depicted in this simplified scheme (3).

In the global nitrogen cycle, nitrogen undergoes continuous oxidation and reduction via distinct biotic and abiotic pathways in soil and marine environments (1). This cycle (Fig. 1) manifests as a dynamic equilibrium between the most oxidized form of nitrogen, nitrate (NO_3_^-^), and the most reduced form, ammonium (NH_4_^+^) (2, 3). Because atmospheric nitrogen (N_2_) is mostly metabolically unavailable, the majority of living organisms rely on the few microbes capable of fixing nitrogen into its various usable forms. Fixed nitrogen is particularly important in soil for utilization by agricultural crops (4, 5). Unfortunately, while the overall negative charge of soil results in the beneficial retention of positively charged NH_4_^+^, negatively charged NO_3_^-^ and nitrite (NO_2_^-^) are readily lost from terrestrial ecosystems (6, 7).

The dissimilatory nitrate reduction to ammonium (DNRA) pathway converts NO_3_^-^ to NH_4_^+^, bypassing the formation of N_2_ (e.g., during the denitrification and anammox pathways) and retaining nitrogen in the soil ecosystem (1, 6, 8). The DNRA pathway is particularly significant in anaerobic environments, where it helps to sustain soil fertility and prevent nitrogen loss that can occur through the denitrification pathway during anaerobic respiration (2, 9). DNRA involves two enzymatic reactions, with nitrate reductase first reducing NO_3_^-^ to NO_2_^-^ and then cytochrome *c* nitrite reductase (NrfA) reducing NO_2_^-^ directly to NH_4_^+^ (9, 10). NrfA performs this six-electron, eight-proton reduction of NO_2_^-^ with incredible efficiency (11, 12), with the specific activity of some NrfAs surpassing 1,000 mol NO_2_ s^–1^ per mol NrfA (13–15). This high turnover rate underscores the enzyme’s remarkable catalytic ability and its vital role in nitrogen transformation within soil environments.

Step 1. Nitrate reduction to nitrite


$$NO_{3}^{-}+\;2e^{-}+2H^{+}\;\overset{\begin{array}{c} nitrate\;reductase \end{array}}{\to }\;NO_{2}^{-}+\;H_{2}O$$

Step 2. Nitrite reduction to ammonium


$$NO_{2}^{-}+6e^{-}+8H^{+}\overset{\begin{array}{c} nitrite\;reductase \end{array}}{\to }\;NH_{4}^{+}+2H_{2}O$$

NrfA relies on a quinol oxidase redox partner to help it efficiently carry out the reaction shown in step 2 above (10). In most organisms, this partner is either NrfB or NrfH (11). NrfB, a soluble, periplasmic protein, attaches to the membrane-bound NrfCD complex to shuttle electrons from the quinol pool in the inner membrane to NrfA (12). In other organisms, however, the function of the NrfBCD complex is replaced by NrfH, which contains four *c*-type hemes and a helical membrane anchor (16). These redox partners will be discussed in more detail later in the review.

**Fig 2:**
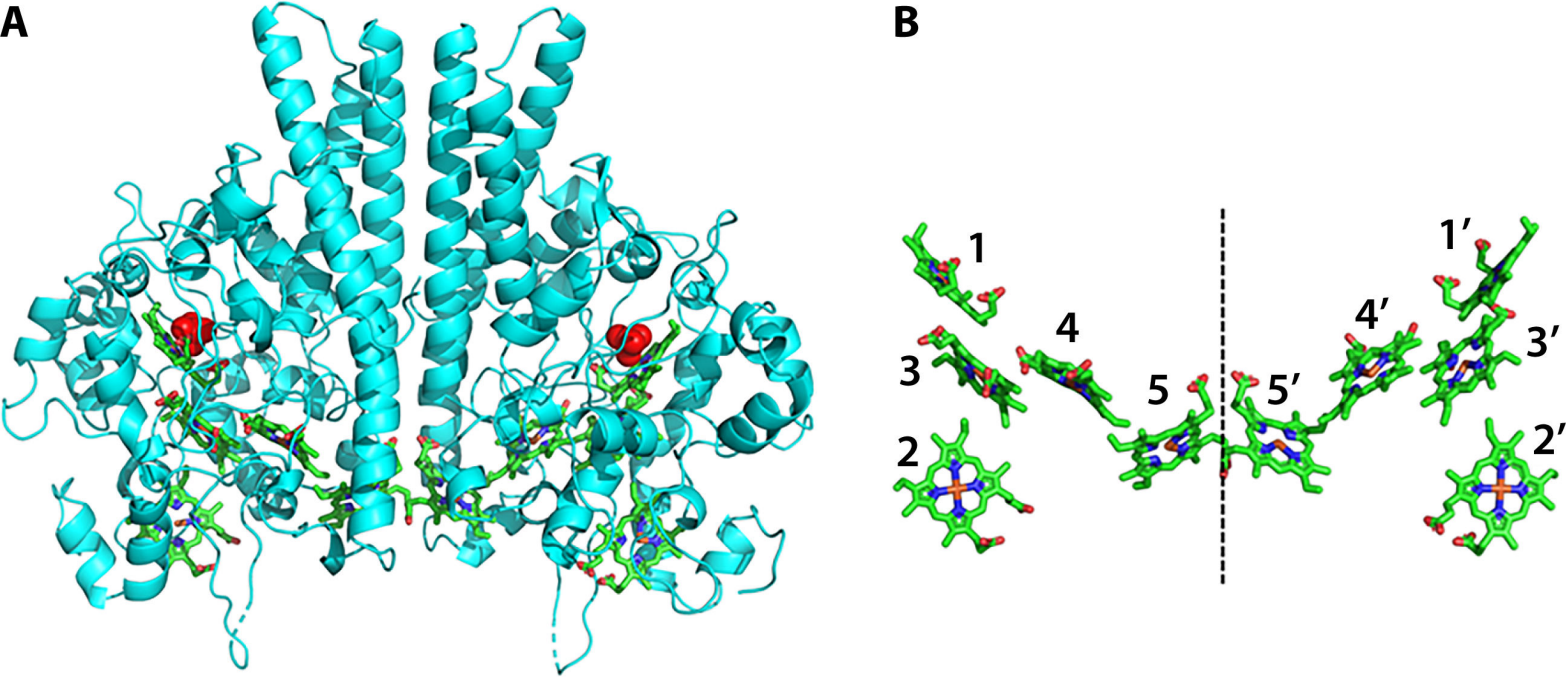
(**A**) Crystal structure of *Geobacter lovleyi* NrfA dimer as a ribbon structure (PDB: 6V0A). The 10 *c*-type hemes (five per monomer) are covalently attached to the protein via two thioether linkages. A sulfate molecule from the crystallization matrix is coordinated to each active site and is shown in red. (**B**) Heme distribution in the NrfA dimer using the same orientation seen in panel A. The heme 5 – heme 5′ edge-edge distance across the interface is 5.2 Å (Fe-Fe distance 11.1 Å). Hemes 2 and 3 shuttle electrons to the active site, while hemes 4 and 5 act as storage units. Hemes 2–5 bind to CXXCH motifs, and heme 1 is bound to a CXXCK motif.

To date, all NrfA proteins crystallize as dimers, regardless of their species of origin (15, 17–23) (Fig. 2A), even though dynamic light scattering studies suggest differing dimerization behaviors in solution for some NrfAs (15, 23, 24). This observation raises the following questions: (i) do some NrfA proteins exist *in situ* as monomers, despite dimerization during crystallization, and/or (ii) are redox partners necessary for physiologically essential dimerization in some organisms? The crystal structure of the NrfAH complex exhibits a [NrfA]:[NrfH] ratio of 2:1 (21). NrfA and NrfB are believed to form complexes in configurations of either NrfB_1_NrfA_1_ or NrfB_2_NrfA_2_ (24, 25). In this review, we compare and contrast the structure, function, and regulation of bacterial cytochrome *c* nitrite reductases and their associated redox partner.

This review is organized into three parts. Part I addresses the evolution and homology of NrfH and NrfA as nitrite reducers in gram-negative bacterial systems. Part II covers the expression of each protein and its regulation as parts of an operon and in response to cellular stressors. Part III details the unique systems involved in the maturation of the cytochrome *c* nitrite reductase system as they relate to their hosts.

## NrfA STRUCTURE AND FUNCTION

NrfA proteins among different bacterial subclasses share very low sequence identities (12). Despite the low sequence identity among NrfA proteins and the use of unique redox partners, however, each NrfA complex performs an identical reduction reaction (12). Here, we focus on what is known about the evolution and homology of NrfA. The NrfA monomer is a soluble, periplasmic, pentaheme *c*-type cytochrome. The body of the protein is globular and primarily made of alpha helices that house the five hemes in an orientation that can be roughly described as Y-shaped (15) (Fig. 2B). Hemes 2 and 3 work in concert to shuttle electrons to the active site from the redox partner, while hemes 4 and 5 are believed to act as storage units in case electrons become limiting (26). These four hemes are all bound to canonical CXXCH motifs and are six-coordinate, *bis*-His ligated. The catalytic site (heme 1) is usually bound to a CXXCK motif with a proximal lysine ligation (12, 27) and is five-coordinate, allowing for facile substrate access to the heme iron (18, 19, 28–31). There are, however, a few NrfAs that utilize CXXCH heme-binding motifs for each of its five hemes, discussed further in “Maturation systems of the NrfHA complex,” below.

**Fig 3:**
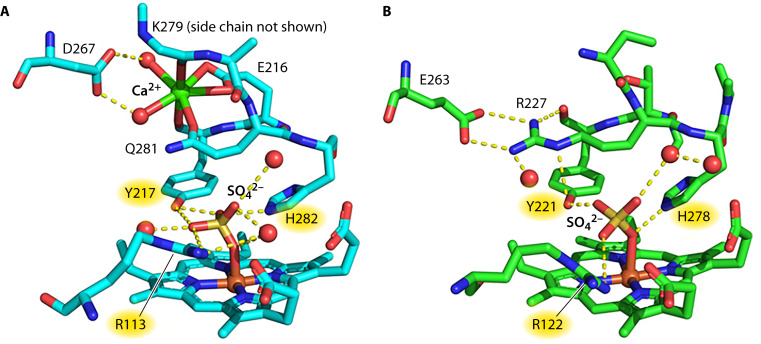
Active-site structure of (**A**) *Sulfurospirillum deleyianum* NrfA and (**B**) *G. lovleyi* NrfA. *S. deleyianum*’s NrfA is a close homolog of the *G. lovleyi* NrfA. The labels of the catalytically relevant second-sphere residues (tyrosine, arginine, and histidine) are highlighted in yellow. The figure is adapted from reference 15.

Near the active site heme are three highly conserved second-sphere amino acid residues, arginine, tyrosine, and histidine (Fig. 3). These amino acids are not directly coordinated to the active site heme, but are critical for the reduction of NO_2_^-^ to NH_4_^+^ (32). These residues surround the substrate and have been shown to create electrostatic interactions with substrate homologs, including the sulfate shown in Fig. 2 (15, 18, 33). With the support of computational calculations, these residues are thought to be involved in several critical roles for catalysis. Through charged interactions with the substrate, they likely guide NO_2_^-^ to the active site and aid binding to the heme iron (18, 20). They are also hypothesized to activate the substrate, first by weakening the N-O bond at the beginning of catalysis, and then by transferring protons to the intermediates throughout the reaction (32, 34).

The most obvious divergence between NrfA proteins from different organisms may be their active site structures. Most NrfA structures solved to date contain a calcium ion near the active site heme that is necessary for activity (13) (Fig. 3A). This calcium ion is hypothesized to work in tandem with the second-sphere residues to help deliver protons to the active site, orient the incoming substrate, and/or help stabilize the appropriate orientation of active site residues for catalysis (10, 20, 32, 34–36). Recent crystal structures of NrfA from *Geobacter* species, however, show an arginine residue where the calcium ion is located in other solved NrfA structures (15, 23) (Fig. 3B). Through phylogenetic analysis, Campeciño et al. (15) discovered this arginine substitution has evolved at least four separate times, with most of the organisms belonging to γ- and δ-proteobacteria or actinobacteria. The presence of arginine was found to be required for enzyme function and likely serves the same function as the calcium ion (15, 23). The four separate emergences of the arginine substitution may signify that either arginine is more efficient than calcium at its function(s) during nitrite reduction or that there was a selective pressure to maintain the function of the calcium ion by organisms living in calcium-depleted environments.

Other proteins related to NrfA can also reduce nitrite to ammonium. A class of nitrite reductase known as NiR performs an identical reaction to NrfA but uses an eight heme-*c* system. Protein sequence alignment shows that the octaheme and pentaheme nitrite reductases are homologous and both have a CXXCK heme-binding motif at the active site (36, 37). The most studied of these octaheme proteins is from *Thioalkalivibrio nitratireducens* (37, 38). It appears that five of the eight *c*-type hemes found in TvNiR and *Geobacter sulfurreducens* (Gs) NiR are conserved in NrfA, and that TvNiR coordinates a Ca^2+^ ion that is necessary for activity near the active site, similar to many NrfA homologs. There is evidence that the pentaheme system evolved first and then fused with a triheme cytochrome *c*, giving rise to the octaheme enzyme with heme 4 of the octaheme (analogous to heme 1 of NrfA) acting as the active site (10, 39, 40).

There are three significant differences between pentaheme NrfA and octaheme NiR besides heme content. First, NiR forms a highly stable hexamer in solution, which is likely necessary for optimal activity. Second, the octaheme appears to be more pH stable and have a higher activity than its pentaheme counterpart (37, 41). Third, the active site second-sphere tyrosine is covalently linked to a cysteine residue which is hypothesized to make the tyrosine a better proton donor (42). Interestingly, *T. nitratireducens* and some of the other organisms with octaheme nitrite reductases also contain a pentaheme NrfA in their genome. The physiological reason for organisms to contain both types of nitrite-reducing enzymes remains unclear.

## EVOLUTION AND HOMOLOGY OF NrfA’S REDOX PARTNERS

The redox partners of NrfA are organism dependent. The primary role of these partners is to transfer electrons from the quinol pool, located in the inner membrane, out to NrfA in the periplasm. Organisms use one of three known redox partners: (i) NrfH (which interacts directly with NrfA and the quinol pool), (ii) CymA (which is a homolog of NrfH and interacts directly with the quinol pool but may or may not interact directly with NrfA; this mechanism is currently unknown), and (iii) NrfB (which interacts directly with NrfA, but works in concert with NrfCD to get electrons from the quinol pool).

### NrfB redox partner

**Fig 4:**
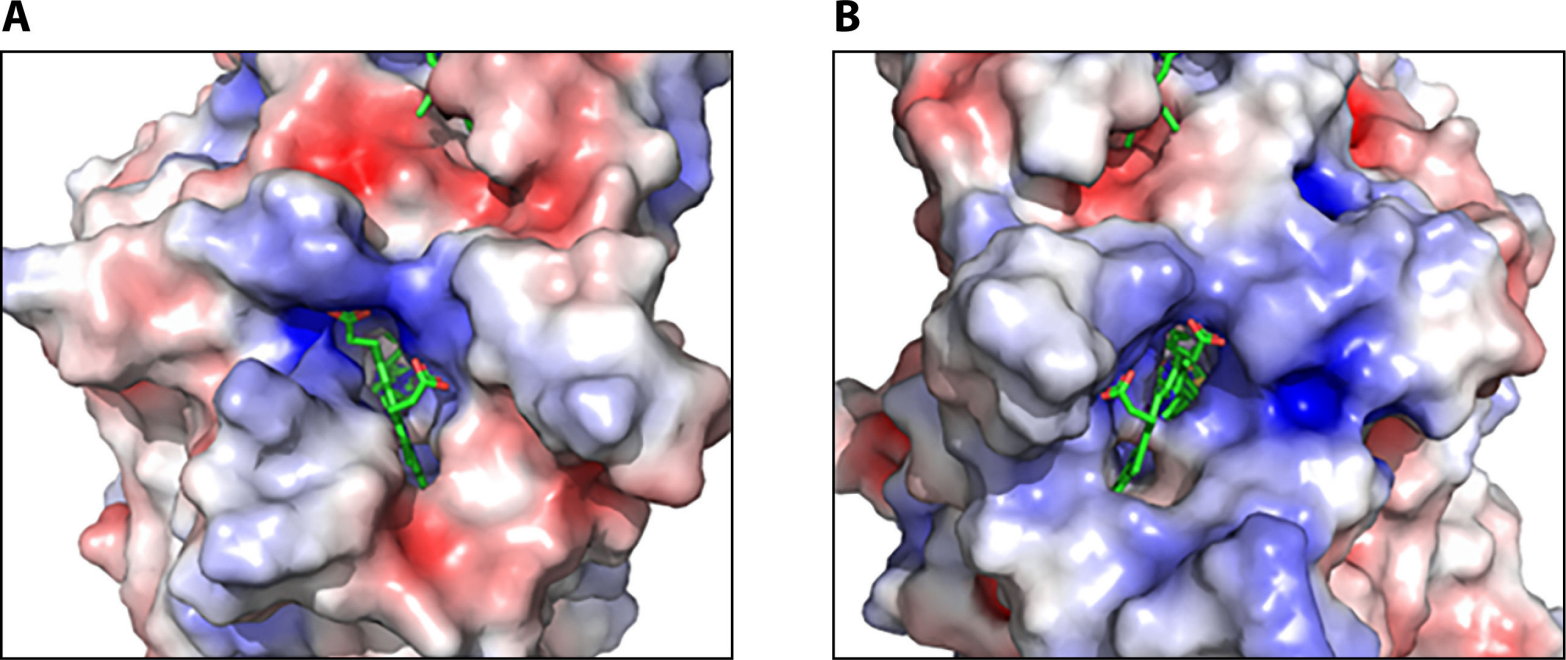
Electrostatic simulation of the surface of NrfA near heme 2 for (**A**) *E. coli* (redox partner: NrfB) and (**B**) *Wolinella succinogenes* (redox partner: NrfH) (19). *E. coli* shows a smaller positive electropositive patch near heme 2, while *W. succinogenes* appears to contain a larger positive patch. The size of the patches may correlate with the binding strength of their respective redox partners.

NrfB, found in γ-proteobacteria (18) such as *Haemophilus influenzae*, *Salmonella enterica, Shigella boydii,* and *Escherichia coli,* is a ~ 20 kDa periplasmic cytochrome *c* protein with five low-spin, *bis*-His hemes (43). Based on crystallographic data and pull-down assays, NrfB binds transiently to NrfA via a weak electropositive charge on the surface of NrfA (18, 19, 24, 25, 43) (Fig. 4A). This positive patch near heme 2 of NrfA also has a conserved group of seven residues (PEFAKGK, PDB:1GU6) that may constitute part of the interaction surface for NrfB (19, 24). Interestingly, all of the NrfA proteins known to date that bind NrfB are calcium-dependent homologs (15).

**Fig 5:**
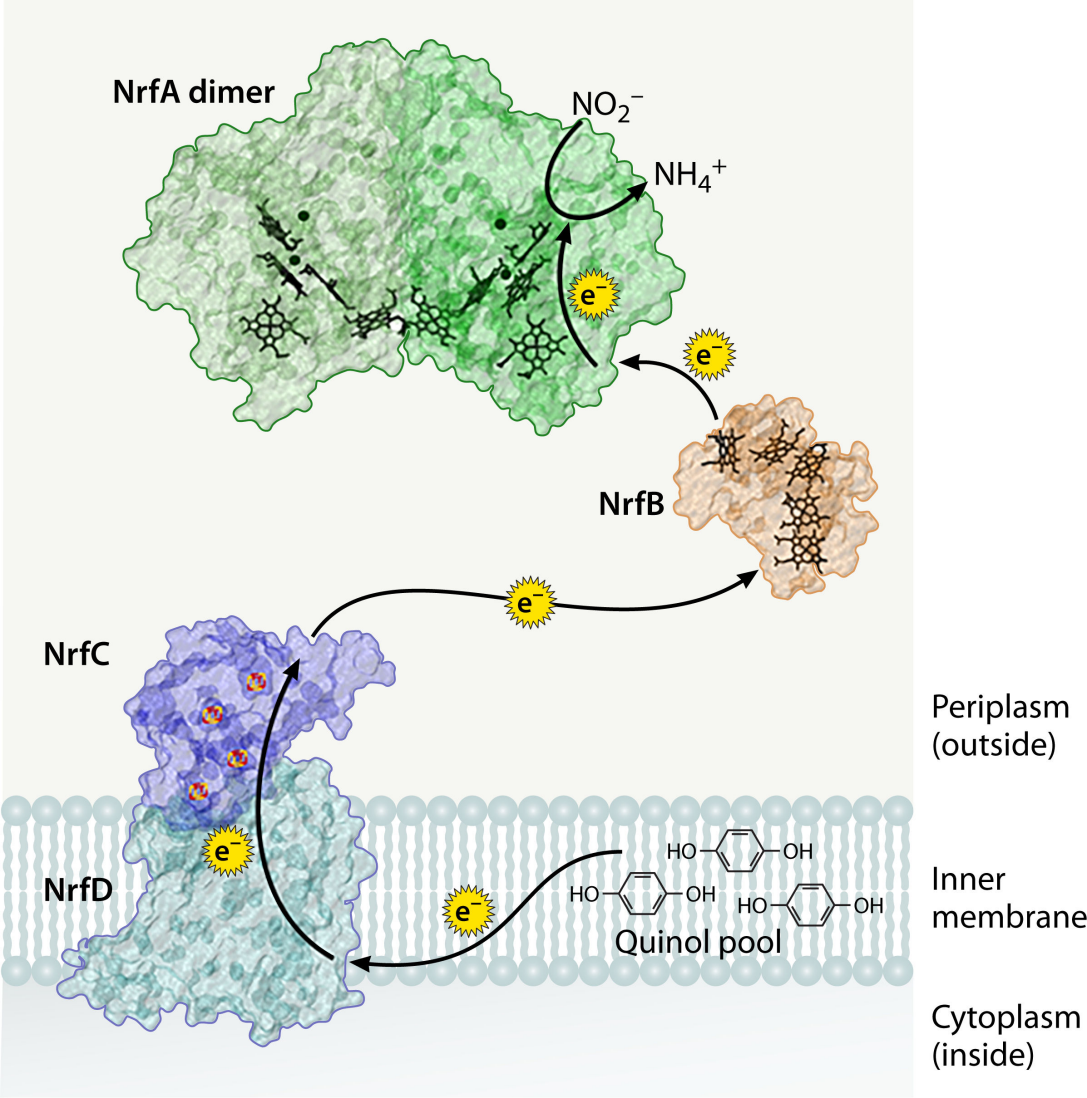
Theoretical flow of electrons between the membrane-bound NrfCD complex and the soluble redox partners NrfB and NrfA. The amino acid sequences from *E. coli* were used to simulate the NrfCD (CAD6019584.1 and CAD6019577.1, respectively) protein structures using Alphafold3 (49). The NrfA and NrfB structures are based on their crystal structures from *E. coli* (PDB: 1GU6 and 2OZY, respectively) (10) and rendered on Visual Molecular Dynamics (VMD). The dark green circles in NrfA are Ca^2+^ ions, the black porphyrin rings in NrfA and NrfB are heme *c*, and the yellow and orange cubes on NrfC are 4Fe-4S clusters.

NrfB obtains electrons from the membrane-bound NrfCD complex before transferring the reducing equivalents to NrfA (Fig. 5). NrfD (~37 kDa) is an integral membrane quinol oxidase with eight predicted transmembrane domains (44, 45), and NrfC (~25 kDa) is a soluble protein with four predicted [4Fe-4S] clusters that is believed to stay in complex with NrfD during electron transfer (46). NrfD is assumed to bind to NrfC in a 1:1 ratio based on homology to the structurally characterized PsrBC complex, which is involved in polysulfide reduction in *Wolinella succinogenes* and *Thermus thermophilus* (47, 48). Due to the transient binding of NrfB to NrfA and the solubility of three of the four proteins in the complex, the NrfABCD complex may form only transiently depending on the needs of the organism (19, 24, 43).

### CymA redox partner

**Fig 6:**
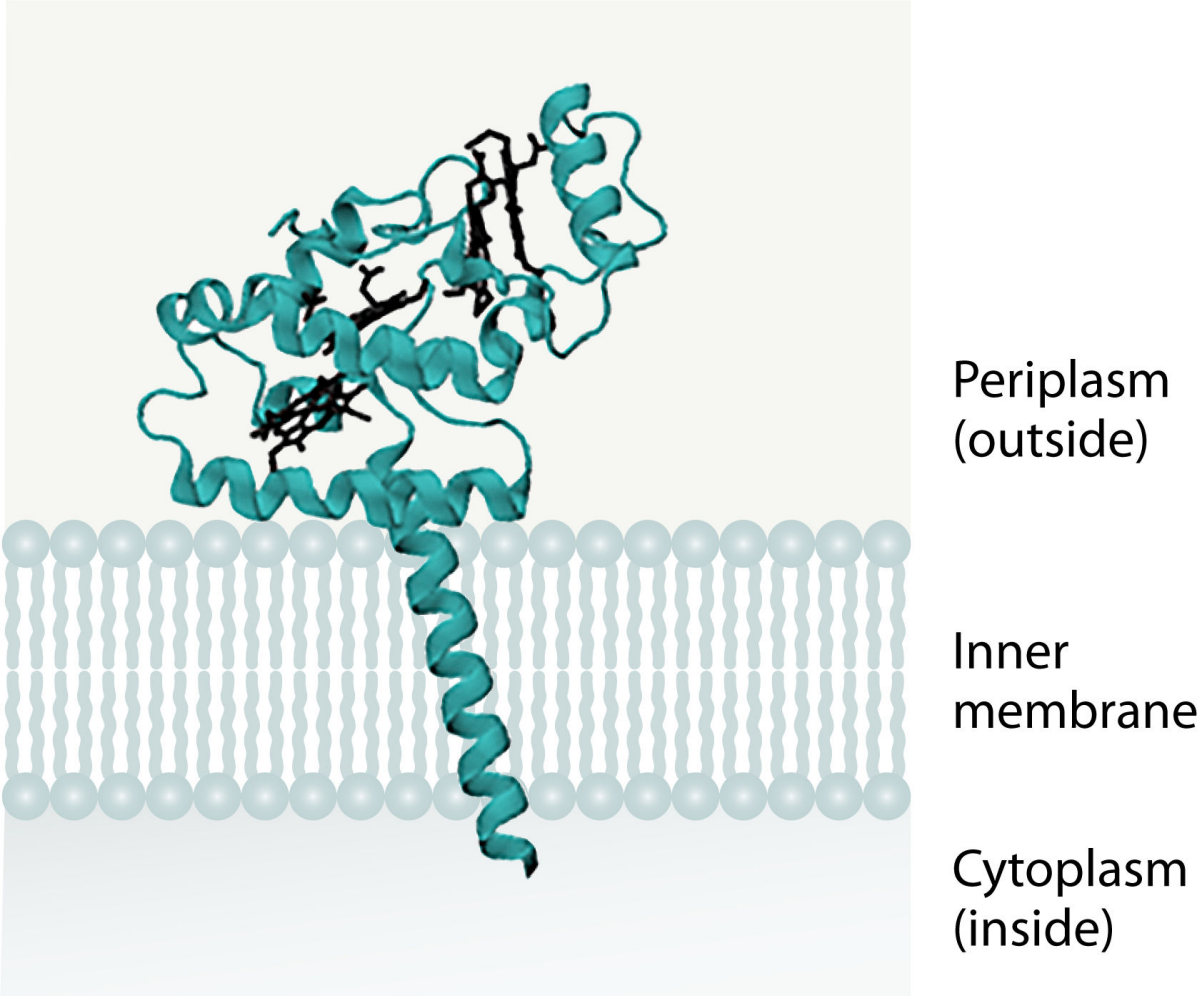
Simulated structure of CymA (WP_011074189.1) from *Shewanella oneidensis* using Alphafold3 (49).

CymA is a 21 kDa membrane-bound cytochrome *c* in the NrfH/NapC protein family with four low-spin hemes (all bound to the canonical CXXCH heme motif) and a transmembrane anchoring helix (50) (Fig. 6). This quinol oxidase has thus far only been found in *Shewanella* (51), a γ-proteobacterial genus, and it is integral to many cellular processes in this organism. Among other things, CymA is involved in the reduction of metal oxides, nitrate, nitrite, dimethyl sulfoxide (DMSO), and fumarate (52, 53). It is unclear whether CymA interacts directly with the terminal reductase enzymes, interacts with small periplasmic cytochromes that act as intermediate electron transfer agents, or if it can do a combination of these duties. This uncertainty extends to its interaction with NrfA. CymA is required for nitrite reduction in *Shewanella* and is therefore involved in electron transfer to NrfA, but it is unknown if CymA interacts directly with NrfA (like NrfB and NrfH) or if there are intermediary proteins between CymA and NrfA (for example, NrfB is the intermediary between NrfA and the NrfCD complex). The variable interactions of CymA may be attributed, at least in part, to a single aspartate residue on the surface of CymA that appears to be necessary for complex formation with its different redox partners in the nitrate and nitrite reduction pathways (54).

CymA’s versatility has enabled it to replace other proteins in the *Shewanella* genome. Although CymA is in the NrfH protein family and would therefore be the expected original redox partner for nitrite reduction, *Shewanella* may have originally used NrfBCD as a quinol oxidation complex. Truncated versions of these proteins have been found in the *Shewanella oneidensis* genome, indicating a shared evolutionary history (55). Simultaneously, and paradoxically, *Shewanella* species may also be moving toward replacing CymA in some of its pathways with dedicated redox partners. Some species do not require it for nitrate or fumarate respiration, likely due to gene duplication and divergence of the dedicated pathway genes (56).

### NrfH redox partner

**Fig 7:**
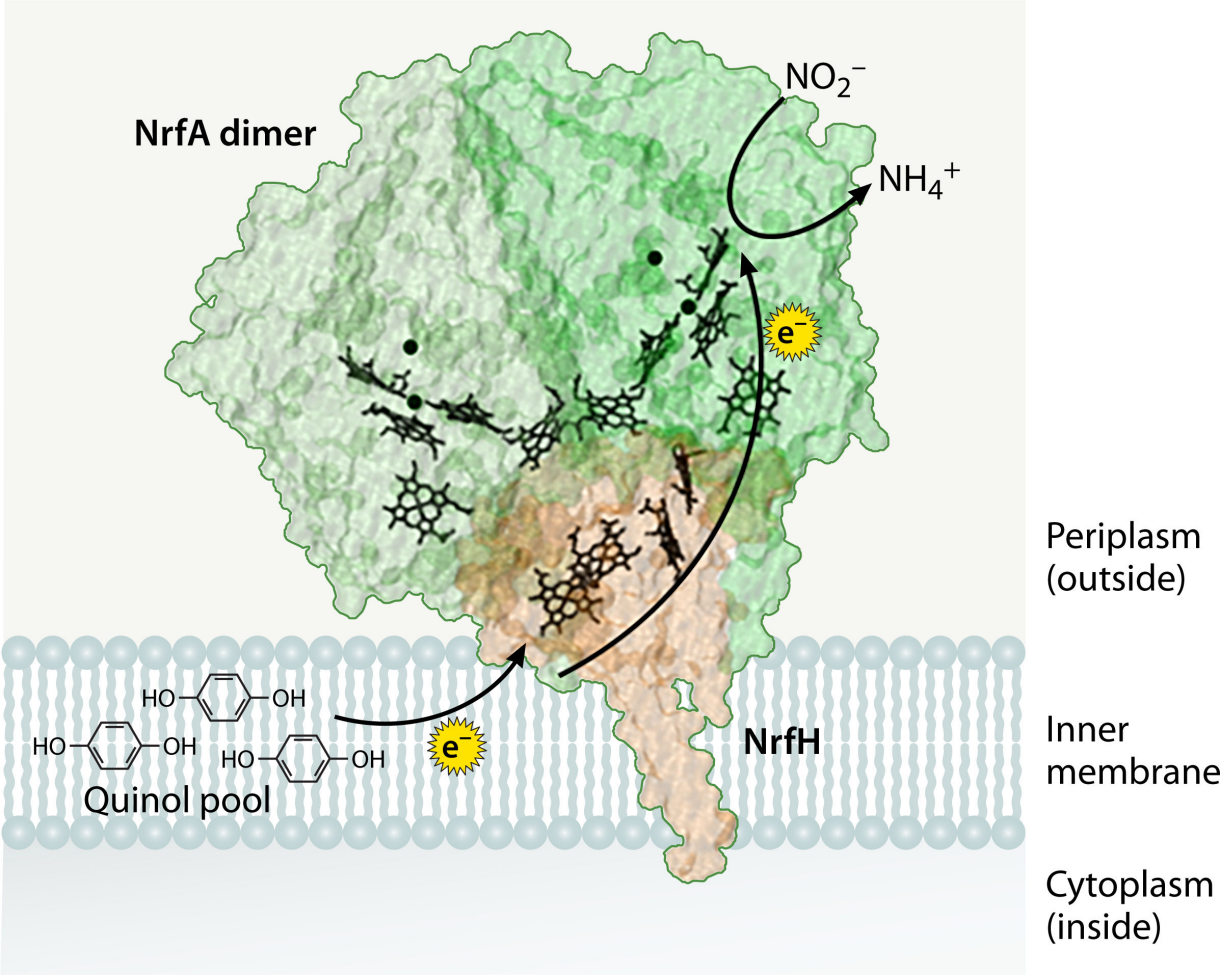
NrfH (orange) crystallized from *Desulfovibrio vulgaris* in complex with an NrfA dimer (green) (PDB: 2J7A) (21). This structure was simulated using Visual Molecular Dynamics (VMD). The dark green circles in NrfA are Ca^2+^ ions, and the black porphyrin rings in NrfA and NrfH are heme *c*.

The NrfH redox partner (Fig. 7) is most commonly present in δ- and ε-proteobacteria (18). As previously mentioned, NrfH belongs to the NrfH/NapC quinol dehydrogenase family of proteins (16). This protein family is composed of roughly 30 proteins, including the known electron donors NrfH, NapC, NirT, DmsC, DorC, CymA, and TorC (57–59), which are each involved in separate pathways. These proteins tend to utilize four or five *c*-type heme cofactors bound to hydrophilic regions of the protein, with NrfH specifically binding four six-coordinate hemes (21, 29). NrfH contains a hydrophobic domain at the N-terminus that is used to anchor it into the inner membrane of its gram-negative bacterial host (60). NrfH shuttles electrons from the quinol pool to its NrfA redox partner via a very tight interaction (16, 61–63), and in fact, these two proteins frequently purify as a complex (16, 21, 29, 64). This may be due, at least in part, to the more prominent electropositive patch seen on the electron entry site of NrfA homologs that bind NrfH rather than NrfB (Fig. 4B).

NrfH is one of the redox partners needed for the second step of the DNRA pathway, nitrite reduction, and NapC is one of the redox partners needed for the first step, nitrate reduction (65). Interestingly, although NrfH and its structural homolog NapC perform unique roles, the two proteins have not yet been found to function in the same organism (12, 61, 63, 66, 67). The reason for their organismal separation is not known, and the possibility that they can functionally substitute for one another appears to be unlikely (43, 63). NapC and NrfH have different heme-*c* ligation patterns (61), which is most likely a result of their different redox functions. Organisms that do not contain NrfH but still rely on nitrate respiration likely contain an NrfBCD complex instead, as is the case for *E. coli* (19). The replacement of NapC in NrfH-containing nitrate-respiring organisms, however, is less understood (63), although nitrite respiration continues unimpaired and therefore likely relies on a similar enzyme substitution as the one for NrfH. The current hypothesis is that another protein in the *nap* operon assumes the responsibility of quinol oxidation (68, 69), a reasonable hypothesis given that this operon can be particularly complex and varied among different species.

## Nrf PROTEIN MATURATION SYSTEMS

Cytochrome *c* maturation systems involved in Nrf protein synthesis appear to be limited to systems I and II. The maturation process occurs in the periplasm, which means both the polypeptide and the heme cofactors must be transported across the inner membrane for attachment. These systems therefore contain heme translocators, heme chaperones, and heme lyases to produce mature cytochromes. Organisms that express CXXCK-containing NrfA proteins must also express a heme chaperone system that can recognize this unique heme-binding motif, discussed in more detail below.

### Maturation systems of the NrfABCD complex

Organisms such as *E. coli* that express NrfABCD use the system I cytochrome *c* heme maturation (Ccm) apparatus (12, 70–75). The system I *ccm* operon contains nine genes, *ccmABCDEFGHI*, which are necessary for the formation of active nitrite reduction complexes. This system of proteins works together to shuttle hemes to the periplasm and insert the hemes into the appropriate apocytochrome. Due to the heavy involvement of cytochromes in anaerobic cell processes, the *ccm* operon is significantly upregulated under anaerobic conditions.

**Fig 8:**
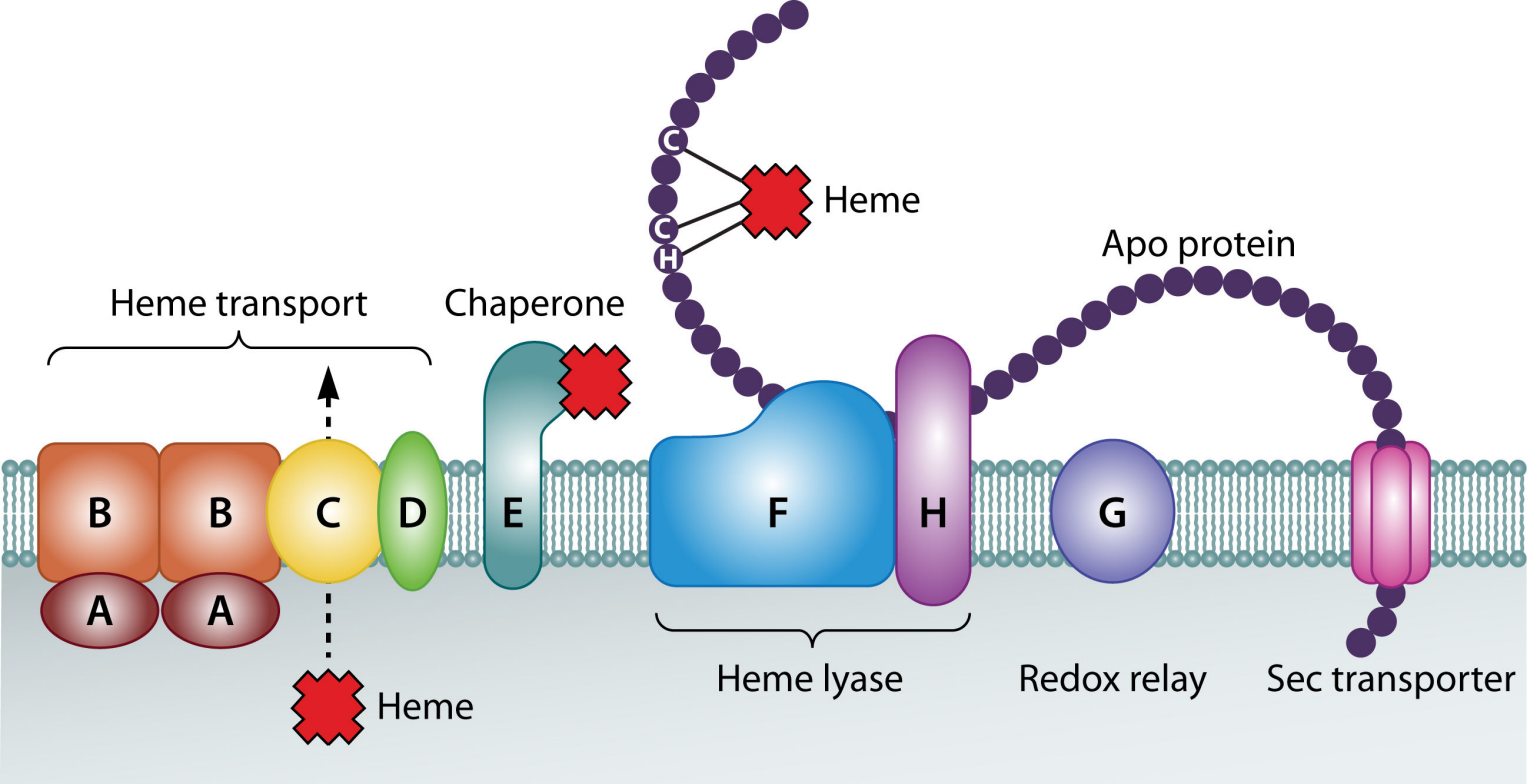
System I cytochrome *c* maturation system proteins with an apo-protein depicted that is being fed through the system. CcmI is not shown as it is commonly fused with CcmH. Figure adapted from reference 83.

First, the membrane complex CcmABCD (76, 77) translocates heme from the cytoplasm into the periplasm (Fig. 8) where CcmAB forms a unique ABC transporter (78, 79). CcmB is an integral membrane protein that anchors the soluble CcmA protein to the complex. The membrane protein CcmC moves heme *b* across the membrane near the CcmBC interface (76). CcmC contains a conserved WXWD heme-handling motif required for stereospecifically carrying and subsequently attaching hemes to the chaperone protein CcmE (80–82).

Next in this reaction pathway is the transfer of heme from CcmC to CcmE. For the heme exchange, CcmC and CcmE interact directly in a stable CcmC-heme-CcmE complex (81, 84). Holo-CcmE is released from the CcmCDE complex upon CcmAB binding to CcmD and subsequent ATP hydrolysis by CcmA (78, 79, 81, 85–87). CcmE has a single anchoring transmembrane helix and a beta-barrel fold (88–91) with a conserved histidine or cysteine residue that covalently ligates the heme cargo (92–96) and then delivers the heme to the heme lyase CcmF (97–101). The interaction between CcmE and CcmF is facilitated by three conserved histidines on CcmF that are necessary for complex formation.

Verissimo et al. (99) also showed that CcmE may have a more direct role in heme attachment to apocytochromes than just transferring the cofactor to CcmF. However, because CcmF contains the necessary WXWD heme-handling motif for transferring hemes to apocytochromes, CcmE’s possible role in the heme transfer is unclear (102, 103). Therefore, this interesting covalent attachment and detachment of a heme to its chaperone requires further study.

CcmF is an integral membrane protein with a heme *b* cofactor that is likely used to reduce the hemes from CcmE to aid in attachment to apocytochromes, as only reduced hemes can be transferred to the lyase (85, 104, 105). Because CcmH is necessary for an active heme lyase complex and has been shown experimentally to have reductase activity in addition to the conserved LRCXXC motif for cysteine reduction, CcmH and CcmF likely work together to reduce disulfide bonds on apocytochromes (106–109). These apocytochromes first interact with CcmI (in *E. coli*, CcmH and CcmI are fused and referred to simply as CcmH) (75, 110, 111) and are then fed through the CcmFHI complex which scans the polypeptide for the requisite CX_n_CH heme-binding motif and initiates cofactor binding (75, 83, 112, 113).

Lastly, the heme cofactor is covalently bound via its two vinyl groups to the cysteine residues in the CX_n_CH motif on the polypeptide chain. CcmG, a small, membrane-anchored protein with a tetratricopeptide repeat (TPR) domain and a thioredoxin motif (114, 115), helps reduce any disulfide bonds that may form at the CX_n_CH motif prior to heme binding (116, 117). A variety of reviews are available for additional information on the system I heme *c* maturation system (10, 45, 70–73, 75, 118–126).

The active site of NrfA, however, contains a unique CXXCK heme *c*-binding motif. In *E. coli*, the proteins NrfEFG are required for heme attachment to the CXXCK motif in NrfA (127, 128). NrfE is a paralog of CcmF and is therefore believed to act as a heme lyase that aids in delivering reductant to hemes and is involved in heme attachment to the apocytochrome (75, 127, 129). NrfF is a paralog to the N-terminus of CcmH, and NrfG is a paralog to CcmI (or the C-terminus of CcmH in *E. coli*). Together, the NrfFG complex is a thiol oxidoreductase that reduces the cysteines in the active site CXXCK-binding motif (75, 129). It is unclear which protein is responsible for recognizing the CXXCK motif and attaching heme to the apocytochrome. Interestingly, *S. oneidensis* does not contain the *nrfEFG* genes and does not appear to have a dedicated heme lyase for its NrfA protein; instead, it appears that CcmF, with the aid of CcmI, may be able to ligate hemes to both CXXCH and CXXCK motifs (55, 130).

NrfG and CcmI have TPRs that allow them to bind to the helical C-terminal region of their respective NrfA proteins for heme transfer (55, 131). The *E. coli* NrfA also consists of a six-residue loop following the C-terminal helix that makes it specific for NrfG. Therefore, CcmI from *S. oneidensis* and NrfG from *E. coli* are not interchangeable chaperones.

### Maturation systems of the NrfHA complex

In contrast to NrfABCD, the NrfHA complex uses the system II cytochrome *c* maturation machinery, which can be found in β-, δ-, and ε-proteobacteria, including the genera *Wolinella, Helicobacter, Desulfovibrio,* and *Chlamydomonas*. This system looks different in different organisms. For example, in *Bacillus subtilis*, a well-studied gram-positive bacteria that does not contain the *nrf* operon but utilizes cytochromes, system II consists of ResB, ResC, ResA, and CcdA (132–138). However, *Wolinella succinogenes*, a gram-negative bacteria that uses NrfAH for nitrite reduction, uses proteins CcsA2, CcsA1, NrfI, CcsX, and CcdA (16, 139–144).

The system II heme maturation process contains two main types of proteins, the cytochrome *c* synthases and the thiol oxidoreductases. Cytochrome *c* synthases have several jobs: (i) transporting reduced heme *b* from the cytosol to the periplasm, (ii) recognizing the necessary heme-binding motifs, and (iii) attaching the hemes covalently to the apocytochromes *c*. Synthases include the previously mentioned CcsA2 (CXXCH) (139, 140), CcsA1 (CX_15_CH) (142, 143), NrfI (CXXCK) (139, 141), and ResBC (CXXCH) (135), in addition to the well-studied CcsAB heterodimer (CXXCH) (144, 145) that is homologous to the ResBC complex and CcsA2. The thiol oxidoreductases (ResA, CcdA, CcsX, and CcdA) have the collective job of obtaining electrons from the cytosol and reducing the disulfide bonds on apocytochromes *c* heme-binding motifs, thereby enabling the attachment of the heme cofactor (120).

**Fig 9:**
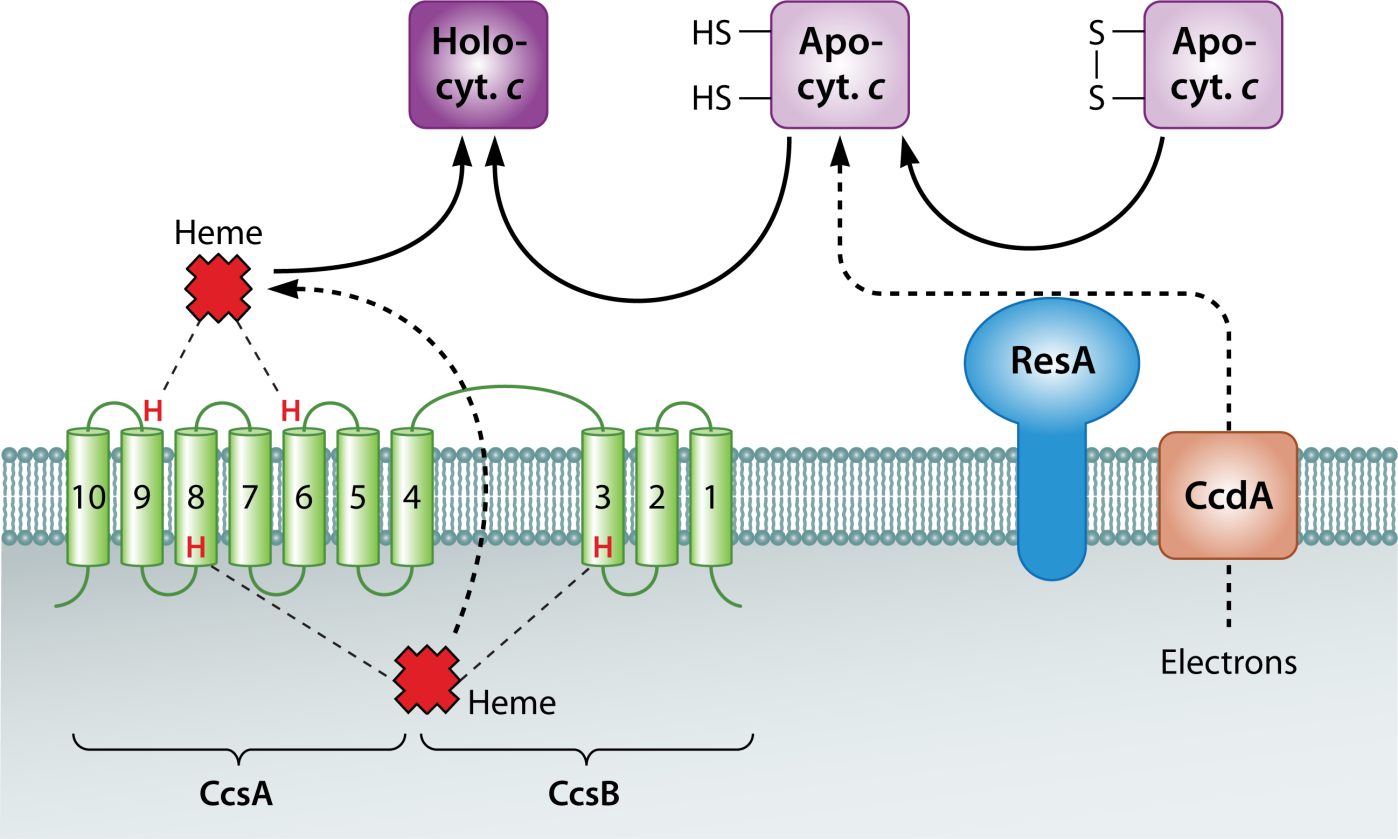
System II cytochrome *c* maturation system proteins showing heme *c* attachment to an apocytochrome. Figure adapted from reference 120.

As an example of what occurs during cytochrome *c* maturation in system II, we will go into more detail about the cytochrome synthase CcsAB, which has both fused and unfused homologs. The CcsAB dimer exhibits a complex structure necessary for cytochrome maturation (146) (Fig. 9). The dimer contains multiple transmembrane (TM) domains, the exact number of which depends on the organism. There are at least six TM segments from CcsA and at least four from CcsB, but there can be as many as 14 TM segments total for the full complex (120). CcsAB is a heme *b* transporter and cytochrome *c* synthase (134, 147). There are two conserved histidines, one on CcsB and one on CcsA, near the cytoplasmic sides of TM3 and TM8 that are necessary for heme transport into the periplasm (147–149). CcsA contains a WWD motif flanked by two conserved histidines on periplasmic loops near TM6 and TM9 that together create an external heme-binding domain (82, 150) that protects the hemes from oxidation (148). The WWD domain is believed to orient the vinyl groups of heme *b* toward the CXXCH-binding motif of the apocytochrome *c* for thioether bond formation (82, 151). In this way, CcsAB has two heme-binding sites that can both be occupied during heme shuttling into the periplasm. When heme occupies the external heme-binding domain, the complex is in an “open” position and is primed for heme attachment to an apocytochrome (147). CcsB may also play a role in orienting the apocytochromes for heme attachment (72). Once the heme is attached to the heme-binding motif, the holocytochrome is released, and CcsAB moves into a “closed” position until another heme is obtained. The CcsAB complex contains a conserved “beta cap” that covers the WWD motif when in the closed position (146, 147). For more information on system II cytochrome maturation, refer to the following reviews: (70, 72, 120, 124, 125, 152).

Organisms that utilize cytochromes with non-canonical heme-binding motifs, such as the CXXCK at the NrfA active site, require maturation machinery that can recognize the unusual motifs. *W. succinogenes* utilizes the NrfI protein, a CcsAB fusion homolog from the operon *nrfHAIJ*, for maturation of the CXXCK active site (16, 141). In addition to the CXXCK motif itself, there are also likely structural features that are necessary for NrfI recognition (153), given that engineered CXXCK motifs were not hemylated by NrfI (139). NrfI from *W. succinogenes* remains the only confirmed protein that recognizes the CXXCK motif in organisms that utilize the system II maturation system, and therefore, it remains unknown how other organisms in this class fulfill this role.

Interestingly, there are some NrfA proteins that have five canonical CXXCH motifs; all use NrfH as the redox partner (12, 27), and therefore they most likely use the system II maturation proteins. For organisms like *Campylobacter jejuni* and *Anaeromyxobacter dehalogenans*, we know that the CXXCH NrfA can perform DNRA without issues. It has not been reported if the active site heme is proximally coordinated by the histidine in the motif in each of these unique NrfAs, or if some use a lysine from elsewhere in the polypeptide chain for the proximal ligation spot (40). Coordination by a lysine that is not part of a CXXCK motif was observed in the octaheme tetrathionate reductase from *S. oneidensis*. Although this protein contains eight CXXCH motifs, the active site heme still coordinates a lysine residue as its axial ligand (12, 40, 154).

## EXPRESSION AND REGULATION

**Fig 10:**
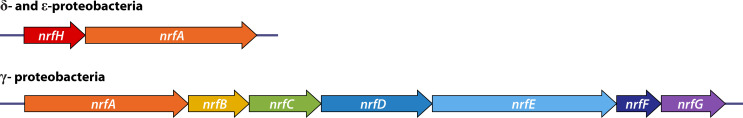
Organization of the *nrfHA* and *nrfABCDEFG* operons, adapted from reference 10.

Both main types of *nrf* operons (*nrfHA* and *nrfABCDEFG*) (Fig. 10) are regulated by the Crp-FNR protein family (155–162). This family includes two large groups of regulators: FNR (the regulator of fumarate and nitrate reduction) and Crp (cyclic AMP receptor protein). FNR and Crp act as both global repressors and global transcription activators that are necessary for multiple respiratory pathways in gram-negative bacteria (163). This family of transcription factors contains both a conserved C-terminal DNA-binding domain that allows it to directly influence expression of respiratory genes and a conserved N-terminal domain for sensing the concentration of the small molecules cAMP, O_2_, and NO (161). In the case of Crp, cAMP must bind to the N-terminal domain to activate the C-terminal-binding domain (161, 164). FNR is instead regulated by the presence of O_2_ and/or NO (165). FNR contains an iron-sulfur (Fe-S) cluster at its N-terminus that degrades in the presence of oxygen. When oxygen is limiting, the Fe-S cluster is stable, and FNR dimerizes to itself and is now able to bind to DNA via its C-terminal end (165). The loss of the Fe-S cluster causes FNR to dissociate into a monomeric state that binds only weakly to DNA (160). Both FNR and Crp are critical for the transition from aerobic to anaerobic growth through the activation of anaerobic processes, including nitrate/nitrite respiration, fumarate respiration, and DMSO respiration (155, 166, 167).

**Fig 11:**
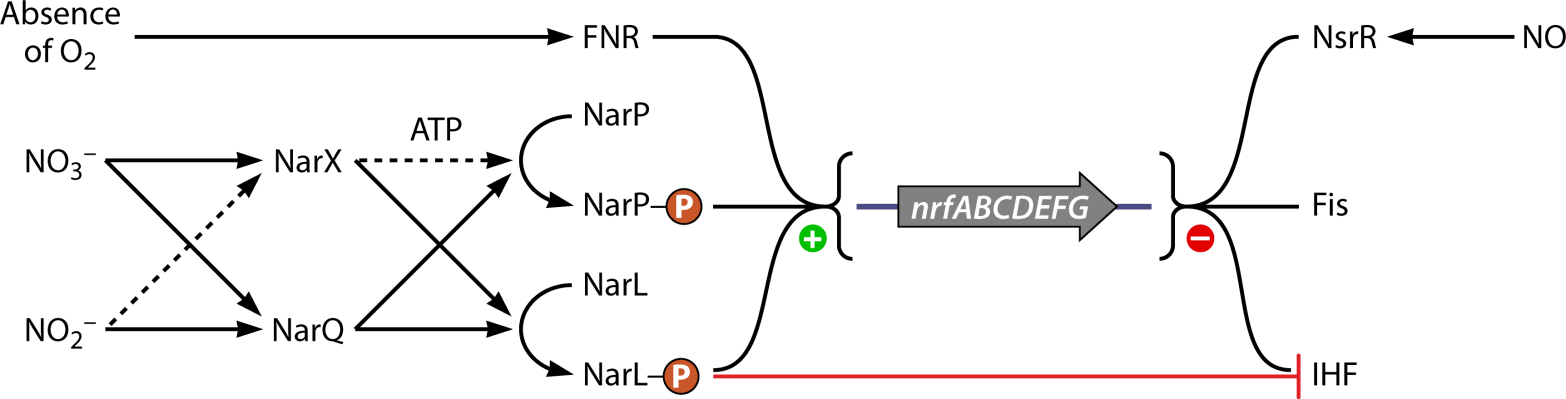
Scheme depicting the regulation of the *nrf* operon. FNR and the NarX-NarL and/or the NarQ-NarP two-component systems upregulate the *nrf* operon in anaerobic and nitrate/nitrite-abundant environments. NsrR is a global repressor that acts on the *nrf* operon, along with the Fis and IHF repressors. NarL can undo IHF repression. The green plus sign next to the operon indicates activation, and the red minus sign indicates repression. The dashed arrows indicate minor/infrequent actions by the proteins, and solid arrows indicate main actions.

Expression of the *nrfABCDEFG* and *nrfHAIJ* operons, however, is affected by more than simply the presence/absence of a single activator. Instead, expression of the *nrf* operon requires an intricately woven network of activators and repressors tied to the Crp-FNR family. We will focus on how the well-studied *E. coli* system regulates nitrite/nitrate respiration. The upregulation of the *nrfABCDEFG* operon is attributed to FNR and the two two-component systems NarX-NarL and NarQ-NarP (168, 169) (Fig. 11). FNR is constitutively expressed, resulting in the cell maintaining low levels of Nrf proteins at all times (160). FNR, however, upregulates nitrate/nitrite reduction in oxygen-limiting environments, while NarX and NarQ (both membrane-bound histidine kinases) upregulate reduction in the presence of nitrate and/or nitrite specifically. NarX and NarQ work in concert with FNR to activate the *nap* and *nrf* operons under anaerobic and high nitrate/nitrite conditions (170). NarX is far more sensitive to nitrate (171), but NarQ recognizes both nitrate and nitrite. Nitrate/nitrite binds to the sensor domain of NarX or NarQ, signaling to these enzymes to phosphorylate the relevant transcription response regulator (NarL and NarP) (163, 169, 172, 173).

Both NarL and NarP recognize and bind to heptameric inverted repeats in the operon promoter regions upstream of the FNR-binding sites (174, 175). Transcription of the *nap* operon, whose gene products reduce nitrate to nitrite, is regulated by the dual action of FNR and NarP (167, 170). Transcription of the *nrf* operon is modulated by the action of FNR, NarP, and NarL. Whereas FNR is strictly required to activate the *nrf* operon, NarL and NarP are important for high expression of the operon (176).

The repression of the *nrf* and *nap* operons is nearly as complex as their activation. Two factors, Fis and IHF, bind to multiple sites on the *nrf* promoter and repress FNR-dependent transcription (176). Interestingly, NarL can displace the IHF repressor, but IHF is unable to displace bound NarL (177, 178). Fis repression cannot be counteracted by FNR, NarL, or NarP activation (176). NsrR is a third repressor of the *nrf* operon that also acts on *nap* (158). NsrR is a global regulator of the reactive nitrogen species response in *E. coli*, and it senses NO directly via a [4Fe-4S] or [2Fe-2S] iron-sulfur cluster (179). Therefore, unless overridden by activators that sense high nitrate/nitrite levels in the cell, NsrR represses both operons (158, 179).

The responses of other organisms to nitrate and nitrite respiration vary widely, and some of the studied systems are summarized in Table 1.

**TABLE 1:** Proteins responsible for the expression levels of Nap and Nrf proteins in the DNRA organisms: *E. coli*, *S. oneidensis*, *W. succinogenes*, and *D. vulgaris*

Organism	Regulator	Senses	Operon	Action	Reference
*E. coli*	NarX	NO_3_^-^	*nrf*, *nap*	Activation	(177, 180, 181)
	NarQ	NO_3_^-^, NO_2_^-^	*nrf, nap*	Activation	(155, 156, 171, 177, 180–182)
	NsrR	NO	*nrf, nap*	Repression	(158, 179, 183, 184)
	FNR	O_2_	*nrf, nap*	Activation	(155, 163, 170, 177, 185–187)
*S. oneidensis*	NarQ-NarP	NO_3_^-^, NO_2_^-^	*nap, nrf*	Activation	(157)
	Crp	cAMP	*nap, nrf*	Activation	(157, 188)
	FNR (EtrA)	O_2_	*nap, nrf*	Mild activation	(189)
*W. succinogenes*	NssA/NssB/NssC	NO_3_^-^, NO, N_2_O	*nap, nrf*	Activation	(159)
*D. vulgaris*	NrfS-NrfR	NO_2_^-^	*nap, nrf*	Activation	(190)

## FINAL PERSPECTIVE

NrfA is notable for its unique functionality, which has evolved in conjunction with species-specific redox partners and independent maturation systems. Interestingly, the redox partner used by NrfA seems to coincide with the type of cytochrome maturation system of the organism, although further research will be needed to ascertain if this apparent relationship is physiologically relevant.

The cytochrome *c* maturation systems involved in Nrf protein synthesis are crucial for the proper functioning of nitrite reduction in various bacteria. These systems, primarily categorized into systems I and II, facilitate the transport and attachment of heme cofactors to their respective apocytochromes in the periplasm. The intricate processes involving heme translocators, chaperones, and lyases ensure the formation of mature and functional cytochromes. Understanding these maturation mechanisms sheds light on the complex biochemistry of bacterial respiration.

The regulation of NrfA varies significantly across species due to its different cellular roles, although its primary role is in anaerobic nitrate respiration (191). In some species like *D. vulgaris*, however, NrfA is used for detoxifying reactive nitrogen species (e.g., NO, hydroxylamine, and hydrogen peroxide) rather than respiration (190, 192), and NrfA may also be involved in sulfite reduction in some organisms (191). Despite these differing regulatory controls, the reaction mechanism of nitrite ammonification is conserved across NrfA variants. The reaction mechanism is also presumably conserved among octaheme nitrite reductases. The presence of these octaheme enzymes, which perform the same reaction as NrfA and coexist with NrfA in the same genome, adds unknown variables to how regulation is managed in DNRA organisms.

The regulation of nrf operons is a complex and finely tuned process involving multiple layers of control by the Crp-FNR protein family and other regulatory elements. The interplay between activators such as FNR and Crp, and repressors such as Fis, IHF, and NsrR, ensures that the expression of these operons is tightly regulated in response to environmental conditions. This intricate regulatory network allows bacteria to efficiently transition between aerobic and anaerobic respiration, optimizing their metabolic processes for survival and growth in varying environments. Understanding these regulatory mechanisms provides valuable insights into bacterial adaptability and the broader implications for microbial ecology and biotechnology.

## Data Availability

The data cited in this minireview are openly available in the references provided.
